# Lack of Impact of *Posidonia oceanica* Leaf Nutrient Enrichment on *Sarpa salpa* Herbivory: Additional Evidence for the Generalist Consumer Behavior of This Cornerstone Mediterranean Herbivore

**DOI:** 10.1371/journal.pone.0168398

**Published:** 2016-12-19

**Authors:** Candela Marco-Méndez, Caitlin Wessel, Whitney Scheffel, Luis Ferrero-Vicente, Yolanda Fernández-Torquemada, Just Cebrián, Kenneth L. Heck, Jose Luis Sánchez-Lizaso

**Affiliations:** 1 Department of Marine Science and Applied Biology, University of Alicante, San Vicente del Raspeig, Alicante, Spain; 2 Marine Research Centre of Santa Pola (CIMAR), Santa Pola City Council, Alicante, Spain; 3 Dauphin Island Sea Lab, Dauphin Island, Alabama, United States of America; 4 Department of Marine Sciences, University of South Alabama, Mobile, Alabama, United States of America; University of Hyogo, JAPAN

## Abstract

The fish *Sarpa salpa* (L.) is one of the main macroherbivores in the western Mediterranean. Through direct and indirect mechanisms, this herbivore can exert significant control on the structure and functional dynamics of seagrass beds and macroalgae. Past research has suggested nutritional quality of their diet influences *S*. *salpa* herbivory, with the fish feeding more intensively and exerting greater top down control on macrophytes with higher internal nutrient contents. However recent findings have questioned this notion and shown that herbivores do not preferentially feed on macrophytes with higher nutrient contents, but rather feed on a wide variety of them with no apparent selectivity. To contribute to this debate, we conducted a field fertilization experiment where we enriched leaves of the seagrass *Posidonia oceanica*, a staple diet for *S*. *salpa*, and examined the response by the herbivore. These responses included quantification of leaf consumption in fertilized and non-fertilized/control plots within the bed, and food choice assays where fertilized and non-fertilized/control leaves were simultaneously offered to the herbivore. Despite the duration of leaf exposure to herbivores (30 days) and abundant schools of *S*. *salpa* observed around the plots, leaf consumption was generally low in the plots examined. Consumption was not higher on fertilized than on non-fertilized leaves. Food choice experiments did not show strong evidence for selectivity of enriched leaves. These results add to a recent body of work reporting a broad generalist feeding behavior by *S*. *salpa* with no clear selectivity for seagrass with higher nutrient content. In concert, this and other studies suggest *S*. *salpa* is often generalist consumers not only dictated by diet nutrient content but by complex interactions between other traits of nutritional quality, habitat heterogeneity within their ample foraging area, and responses to predation risk.

## Introduction

In the Western Mediterranean, seagrass meadows are dominated by *Posidonia oceanica* (L.) Delile [1,2] where the fish *Sarpa salpa* (L.) and the sea urchin *Paracentrotus lividus* (Lam.) are the two main macroherbivores [3]. Herbivory rates on this seagrass species are extremely variable, ranging from 2–57% of *P*. *oceanica* leaf productivity, according to the available literature [4,5]. This variability in estimated herbivory has been suggested to be partly a consequence of the different methods employed for quantification [6]. Through direct methods, *S*. *salpa* has been shown to be an important herbivore accounting for 70% of the total leaf consumption of *P*. *oceanica* [5], although it is also known to ingest large quantities of other macrophytes species [7,8] and to display high spatial and temporal variability in its herbivory pressure [5,9,10]. Some of the factors involved in *S*. *salpa* herbivory include; macrophyte availability and accessibility, habitat heterogeneity, nutritional quality, human pressure on herbivore populations, herbivore recruitment, predation and patterns of movement [11,12,13].

In seagrass ecosystems, the evidence indicates that herbivore distribution is highly influenced by fishing pressure [14]. In this context, marine protected areas (MPA) can alleviate anthropogenic impacts (e.g. overfishing), and enhance local fish recruitment [15]. Indeed, it has been shown that populations of fish, including the herbivore *S*. *salpa*, benefit from fishing protection and tend to concentrate in MPAs, resulting in enhanced grazing pressure [12].

Plant nutritional quality (often expressed as leaf nitrogen content) has also been shown to play a central role in determining herbivore feeding patterns in seagrass systems, suggesting a preference and higher feeding on diets with high nitrogen content [16,17]. Previous studies have suggested that differences in the nutritional quality among seagrass species could result in different levels of herbivory [9,18]. As epiphytes and macroalgae have typically lower C:N ratios than seagrasses [19,20] they are proposed to sustain a comparatively higher herbivory pressure [21] which may be enhanced by nutrient availability. Through experimental studies, it has been shown that nutrient addition can change nutritional properties of seagrass leaves and their epiphytes by increasing nutrient ratios [9], modifying plant defenses [22] or changing the composition of epiphyte assemblages [11]. Indeed, in [9] it was showed that nutrient addition induced changes in both seagrass (enhanced plant N content and leaf growth) and epiphytes (enhanced N content, biomass load and altered species composition) which concurred within higher herbivory pressure by *S*. *salpa*. In a more recent experimental study using moderate, ambient and large scale enrichment plots conducted in *Cymodocea nodosa* (Ucria) beds Ascherson, results also confirmed that increases in nutrient levels can lead to increased grazing bites [23]. This has also been shown in other ecosystems (e.g. salt marshes), throughout experimental approaches, finding that grazers preferentially congregate and graze in areas of marsh with nutrient rich leaves [24].

However, other studies have also found that higher nutrient content did not always lead to increased consumption rates (for a review see [25,26]) as macrophyte-herbivore interactions are dependent on the associated herbivore and macrophytes species. Recent studies have found that not only seagrasses but also other macrophytes species such as *Caulerpa* sp. [7,8,27] may have an important contribution to *S*. *salpa* diet and that final consumption rates and dietary differences may not only be determined by nutritional content but by other macrophytes features [28,29]. The quality or “palatability” of the food is also determined by secondary metabolites, toughness [30], energy contents [31], varying levels of structural carbohydrates (cellulose) affecting food digestibility, and absorption (e.g. [32]). These factors can vary greatly between species and mediate important macrophyte-herbivore interactions [33]. Overall, studies suggest that different herbivore species may be distinctively affected by nutrient conditions, therefore low nutritive value by itself may not always be an effective defense against grazing [34]. Since high nutrient levels and grazing pressure can ultimately depress leaf production, it is important to expand upon the knowledge of what role nutrients serve in driving herbivore feeding preferences. This would help us to better understand the variability of herbivory and how it may affect the distribution of seagrass species.

In the present study, we conducted a field fertilization experiment using the Mediterranean seagrass *P*. *oceanica* to test the effect of nutrient enrichment on seagrass and its potential influence on *S*. *salpa* consumption rates and feeding choices. In order to evaluate whether an increased fish population due to fishing protection can lead to higher herbivory pressure, experiments were carried out in a MPA (Tabarca) and a non-protected area (CIMAR). We hypothesized that fertilization would increase leaf growth rates and decrease C:N ratios, and that *S*. *salpa* will respond by selecting fertilized shoots over controls; displaying higher pressure over these nutritional enriched shoots and recording higher consumption rates in the MPA due to fishing protection.

## Materials and Methods

### Study area

The study was carried out in two locations in the northwest Mediterranean Sea; (1) an MPA in the surrounding waters of Tabarca Island located east of Santa Pola, Alicante, Spain (38°09’52”N, 0°27’46”W) and; (2) a location just offshore of the Centro de Investigación Marina de Santa Pola (CIMAR) in Santa Pola, Alicante, Spain (38°12’27”N, 0°30’17”W). The MPA covers 14.63 km^2^ and was first designated in April of 1986. Within each location, three sites were selected in *Posidonia* beds (4–5 m deep) approximately 50 m apart making a total of six sampling sites with similar bottom characteristics. At each site there were three fertilized plots (512 cm^2^ each) and three non-fertilized (control) plots for a total of nine fertilized and nine control plots in each of the two study locations. Fertilization was conducted for six weeks prior to the start of this study by using slow release nitrogen fertilization rods (Compo) three times in 15–25 day increments. Here we provide some information regarding the composition, the dose recommended for terrestrial plants and the dose we added to plots:

**Composition (per rod):** NPK 13+6+10+ magnesium + micronutrients, 13% total N.
**Dose recommended**: 2 units in 314 cm^2^ (0.0064 units/cm^2^); 1.8 g of rod with 0.5234 g of N in 314 cm^2^; 0.000745 g N/cm^2^. This dose guarantees the nutrients required by the plant during 30 days avoiding detriment to the plant by overdose.
**Dose applied in the experiment**: 20 units/plot in each fertilization time; A total of 60 units/plot in 45 days; 20 units in 512 cm^2^ plot.0.039 units/cm^2^ each time.18 g of rod with 2.34 g of N in 512 cm^2^.
**Dose per fertilization time**: 0.00457 g N/cm^2^ (6 times higher than the dose recommended).
**Total dose during the experiment**:0.01371 g N/cm^2^ (18 times higher than the dose recommended).

Despite we know that there must be an effect of dilution on the amount we added to the plots, we think that it may help to have an idea of the total amount we used. The quantity over exceed 6 times the dose recommended for a first fertilization treatment and 18 times the dose recommended to be added in a month. Also, when we came back every fertilization time, we noticed that part of the rods from the previous treatment remained in the plots, so we consider the dilution was low as well as nutrients release and therefore the fertilization treatment was successful. We deliberately applied a high fertilization dose to mimic large, realistic inputs such as from sewage pipes.

### Permits

All necessary permits were obtained for the described field studies. The sampling carried out in the MPA of Tabarca was permitted by the authorities of the Ministry of Agriculture, Food and Environment (Spain). The sampling carried out in the CIMAR was out of the preserved area and did not require any specific permission. We confirm that that the field studies did not damaged any endangered or protected species.

### Growth rates

For the herbivory experiment, growth rates of *P*. *oceanica* leaves were measured using a method from [35] which was modified from [36]. Within each control/fertilized plot 20–25 shoots were marked by punching two parallel holes in all leaves just above the ligula of the outermost leaf with a hypodermic needle. On day 30 after marking, shoots marked were recovered from each plot (ten shoots per plot, for a total of 90 fertilized and 90 control shoots per location) and elongation and total length of each leaf were measured to the nearest millimeter, as well as any new leaves that did not have a mark scar (new growth). Growth was summed for all leaves within a shoot and estimated in control and fertilized shoots (cm^2^ shoot^-1^ day^-1^).

### Nutrient content (C:N) in leaves and epiphytes

The effect of fertilization on the *P*. *oceanica* shoots was measured in terms of the C:N ratio. Within each of the fertilized and control plots, we haphazardly collected three shoots (a total of 27 fertilized and 27 control shoots per location). First, epiphytes were removed from the leaves and then both leaf material and epiphytes were dried to a constant weight at 60°C and then ground into a powder using a mortar and pestle. The C:N ratios of both the leaves and epiphytes were analyzed using a NA1500 C/N/S analyzer^™^ (Carlo Erba).

### Consumption rates

To determine herbivory, two *Posidonia* shoots were collected from each plot (a total of 18 fertilized and 18 control shoots per location). In the lab leaves were cut to remove prior herbivory marks, their area was measured, and tissues were hole-punched at the base of the leaf to allow estimates of any further growth during the experiment [5]. The shoots were attached to clothes pins using cable ties and were then placed in the plots they were collected from for 30 days. After this period, shoots were re-collected and all leaves were photographed. Leaf area was then measured using Sigma-Scan Pro image software and leaf loss by *S*. *salpa* herbivory was estimated as the difference between initial and final photosynthetic area after growth corrections (cm^2^ shoot^-1^ day^-1^).

### Food choice experiments

Paired food choice experiments were conducted to test *S*. *salpa* preferences for control vs. fertilized leaves. For this purpose *P*. *oceanica* shoots were collected from experimental plots to form tethers [37]. All bite marks were removed with scissors and leaves were hole-punched to control for any growth that might occur during the experimental period [5]. After all the replicates were photographed on a grid to estimate initial surface area by image analyses, they were attached to a sisal line using clips to form tethers. In each location we deployed a tether line with paired replicates (n = 9, control and fertilized shoots tied together), each with three leaves. The tethers were deployed for 30 days and then recollected and photographed to estimate final surface area with Sigma-Scan Pro image software. Leaf loss by *S*. *salpa* herbivory, whose bite marks were easily distinguishable (e.g. [38]), was estimated as the difference between initial and final areas after growth corrections (cm^2^ leaf^-1^ day^-1^).

### Statistical analyses

A three-way ANOVA was used to analyze differences in growth rates, in nutrient content (C:N ratio) of leaves and epiphytes and in leaf loss by consumption due to “Location” (fixed and orthogonal factor with 2 levels), “Site” (random and nested in “location” with 3 levels) and “Treatment” (fixed and orthogonal factor with 2 levels) considering “plot” as a replicate (average value; n = 3 fertilized/control plots per site; 9 fertilized/control plots per location). The model for these analyses was:

X=Mean +Loc +Sit (Loc)+ Tr+Loc X Tr +Tr X Sit (Loc)+Residual

ANOVA assumptions of normality and homogeneity of variance were assessed with the Kolmogorov-Smirnov and Cochran’s C- tests, respectively. When necessary, an appropriate transformation was performed before further analysis. When assumptions were not met, the level of significance was set at 0.01 to reduce the possibility of Type I error [39]. Student-Newman-Keuls post-hoc tests were used to single out significant groupings. The statistical tests were done using PASW Statistics 18 and GMAV 5 software (University of Sydney, Australia).

In order to analyze wether our three-way ANOVA design could guarantee a power analysis is high enough to avoid Type II error (retaining null hypothesis when it is false), we performed a “post-hoc power analysis” [40] with G power software [41].

Results from food choice experiments were tested separately for each location with a Wilcoxon signed-ranks paired test due to lack of normality and homoscedasticity of the data (n = 9 paired replicates).

## Results

### Growth rates

**Fig 1:**
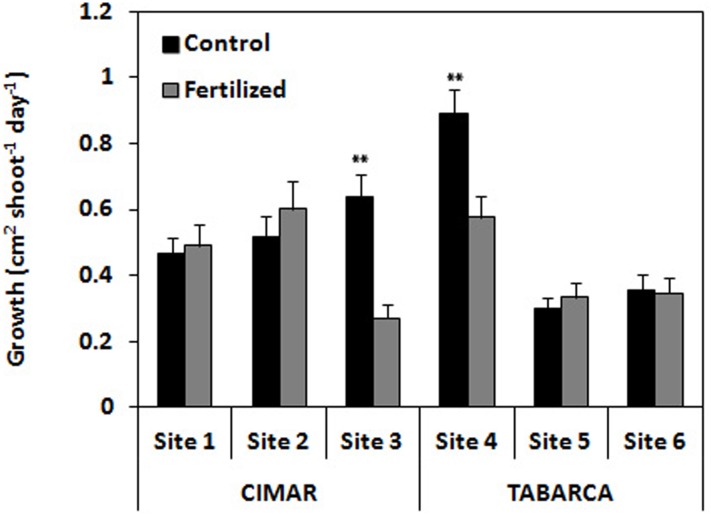
Growth rates recorded in leaves of *P*. *oceanica* at CIMAR and Tabarca in the different sites for control vs. fertilized treatments. Mean ± SE. **p < 0.01.

Significant differences were found for “Site (Loc)” and the interaction “Treatment x Site (Loc)” (three way ANOVA; p < 0.001 and p < 0.01 respectively; Fig 1; Table 1a). At CIMAR, values for the fertilized treatment at site three was significantly lower than in control treatment. At the other sites, values did not differ significantly between treatments (see SNK in Table 1a). At Tabarca, values at site four were significantly higher in control than in fertilized plots, but values did not differ significantly between treatments at the remaining sites (see SNK in Table 1a; S1 Data). The average growth rate recorded at CIMAR for the control treatment was 0.540 ± 0.059 cm^2^ shoot^-1^day^-1^ and for the fertilized treatment 0.454 ± 0.062 cm^2^ shoot^-1^ day^-1^. At Tabarca, the average growth rate recorded for control treatment was 0.514 ± 0.052 cm^2^ shoot^-1^ day^-1^ and for fertilized treatment was 0.419 ± 0.051 cm^2^ shoot^-1^ day^-1^.

**Table 1:** Three way ANOVA results for: (a) growth rates; (b) nutrient content (C:N ratio) in leaves; (c) in epiphytes; and (d) consumption rates showing differences due to investigated factors “Location” (fixed and orthogonal factor with 2 level), “Site” (random and nested in “location” with 3 levels) and “Treatment” (fixed and orthogonal factor with 2 levels) considering “plot” as a replicate. (n = 3 fertilized/control plots per site; 9 fertilized/control plots per location).

Source of variation	a. Growth (cm^2^.shoot^-1^.day^-1^)				b. C:N in leaves				c. C:N in epiphytes				d. Consumption (cm^2^.shoot^-1^.day^-1^)			
	df	MS	F	p	df	MS	F	p	df	MS	F	p	df	MS	F	p
Location (Loc)	1	0.015	0.09	NS	1	7.177	0.02	NS	1	138.322	1.47	NS	1	0.108	0.33	NS
Site Sit (Loc)	4	0.168	14.04	***	4	313.513	4.86	**	4	94.238	14.01	***	4	0.326	0.98	NS
Treatment (Tr)	1	0.081	1.05	NS	1	972.388	20.32	*	1	1.240	0.51	NS	1	0.368	1.09	NS
Loc X Tr	1	0.001	0.01	NS	1	40.016	0.84	NS	1	3.696	1.51	NS	1	0.245	0.72	NS
Tr X Sit (Loc)	4	0.078	6.49	**	4	47.858	0.74	NS	4	2.455	0.36	NS	4	0.339	1.01	NS
Residual	24	0.012			24	64.528			24	6.727			24	0.334		
Total	35				35				35				35			
SNK	CimS1C = CimS1F; CimS2C = CimS2F; CimS3C>CimS3F															
	TabS4C>TabS4F; TabS5C = TabS5F; TabS6C = TabS6F															
Transformation	None				None				None				None			

Significant differences are indicated:

* p < 0.05,

** p < 0.01,

*** p < 0.001,

NS: not significant.

In SNK, significant differences between investigated groups are indicated. Code to read the SNK results: Cim = CIMAR, Tab = Tabarca, S1 = site 1, S2 = site 2, S3 = site 3, S4 = site 4, S5 = site 5, S6 = site 6, C = control/non-fertilized, F = fertilized.

### Nutrient content (C:N) of leaves and epiphytes

**Fig 2:**
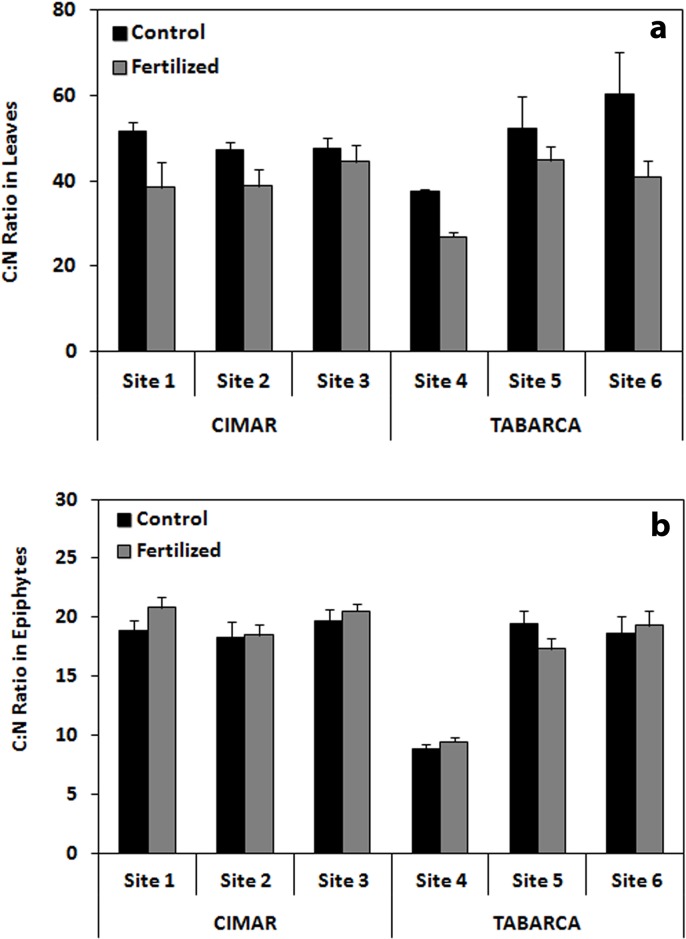
C:N ratio recorded in leaves (a) and epiphytes (b) of *P*. *oceanica* at CIMAR and Tabarca in the different sites for control vs. fertilized treatments. Mean ± SE.

Nutrient content in leaves measured as C:N ratio showed significant differences due to “Site (Loc)” and “Treatment” factors (three way ANOVA; p < 0.01 and p < 0.05 respectively; Fig 2a; Table 1b). Analyses recorded higher values in controls vs. fertilized treatment (see Fig 2a; Table 1b). The average C:N value recorded in the CIMAR control treatment was 48.87 ± 2.18 and for fertilized treatment 40.59 ± 4.55. In Tabarca, the average C:N value recorded for control treatment was 50.09 ± 5.97 and for fertilized treatment was 37.58 ± 2.68 (S2 Data).

In epiphytes, C:N ratio showed significant differences for the “Site (Loc)” factor (three way ANOVA; p < 0.001; Fig 2b; Table 1c). In the CIMAR, significant differences were not found between sites nor between treatments. In Tabarca, values recorded at site five and site six for both treatments were higher than values recorded at site four for both treatments (S3 Data).

### Consumption rates

**Fig 3:**
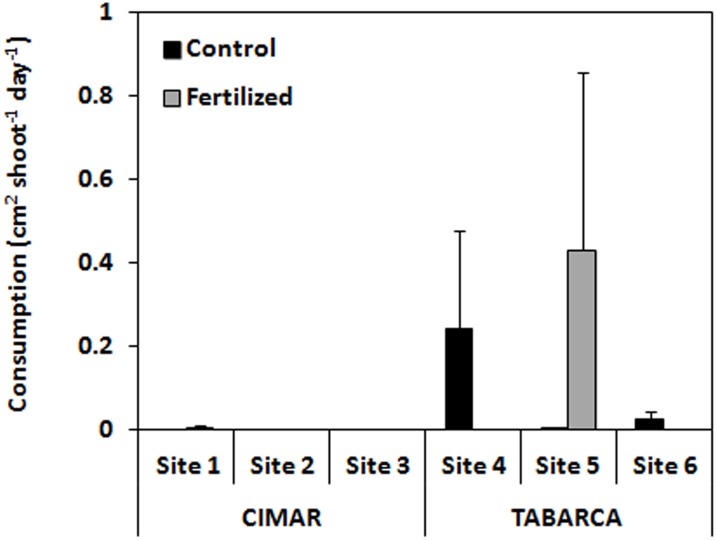
Consumption by *S*. *salpa* recorded at CIMAR and Tabarca in the different sites for control vs. fertilized treatments (cm^2^·shoot^-1^·d^-1^). Mean ± SE.

No significant differences were found in consumption rates for any of the factors investigated (three-way ANOVA; Fig 3; Table 1d). Despite the schools of *S*. *salpa* that frequented areas surrounding our plots at both study locations (Marco-Mendez pers. obs.), values of consumption were low showing a high variability among the different sites. At CIMAR, no consumption was recorded in the control treatments for any site; in the fertilized treatments, average consumption rate recorded was 0.001 ± 0.001 cm^2^ shoot^-1^ day^-1^. At Tabarca, average consumption rates recorded in control treatments was 0.089 ± 0.086 cm^2^ shoot^-1^ day^-1^ and for fertilized treatments 0.143 ± 0.143 cm^2^ shoot^-1^ day ^-1^ (S4 Data).

To assure that we are not committing a Type II error (retaining null hypothesis when it is false) we decided to do a “pooling”, a technique applied to models with random factors to improve their power to detect treatment effect by increasing the denominator degrees of freedom. A common rule is to control Type II error rate by pooling only if p > 0.25 [39] so we pooled the random and nested factor “Site (Loc)” (p = 0.4391). We found no significant difference due to “Location” (p = 0.5743), “Treatment” (p = 0.3563) or the interaction “Location x Treatment” (p = 0.4434.). For this ANOVA design, we performed a “post-hoc power analysis” [40] to test the statistical power. Power analyses for factors, “Location”, “Treatment” and the interaction “Location x Treatment” set for a medium size effect (0.25) and α = 0.05, found a power (1- β error probability) = 0.3019. For a medium or moderate size effect (0.35), the power (1- β error probability) = 0.522. This last value is at least suggestive of a correct decision when accepting our null hypothesis, which in our study is that herbivory by *S*. *salpa* was not higher in the fertilized plots compared to the non-fertilized plots.

### Food choice

**Fig 4:**
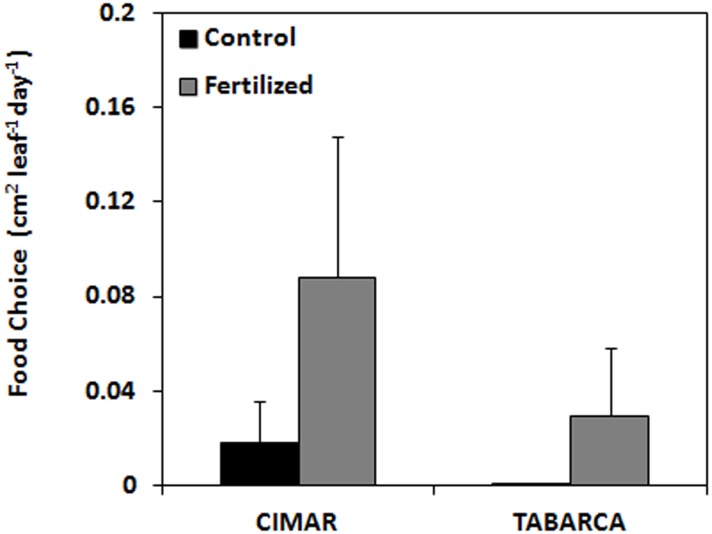
Consumption by *S*. *salpa* of control vs. fertilized leaves of *P*. *oceanica* during paired food preference experiments (cm^2^·leaf^-1^·d^-1^) deployed at Tabarca and CIMAR. Mean ± SE.

Analyses of the consumption recorded by food choice experiment did not detect any significant preference for fertilized vs. control shoots neither at CIMAR (Z = -0.730; p = 0.465; Fig 4) nor at Tabarca (Z = -1.153; p = 0.249; Fig 4). As found in the plots, overall values of consumption detected by tethers were low. Despite the fact that no significant differences were detected, average consumption was higher on fertilized shoots (CIMAR: 0.088 ± 0.059 cm^2^ leaf^-1^ day^-1^; Tabarca: 0.029 ± 0.028 cm^2^ leaf^-1^ day^-1^) than on control shoots (CIMAR: 0.018 ± 0.018 cm^2^ leaf^-1^ day^-1^; Tabarca: 0.001 ± 0.001 cm^2^ leaf^-1^ day^-1^) (S5 Data).

## Discussion

This study suggests that *Sarpa salpa* herbivory did not directly correspond with increased nutritional value of leaves and displayed high spatial variability of consumption in *Posidonia oceanica* meadows. The fertilization experiment did increase the nutritional content of leaves, but this was not an apparent influence neither on *S*. *salpa* herbivory nor leaf growth rates, which contradicts previous findings [9]. Despite schools of *S*. *salpa* frequently observed around the plots (Marco-Méndez pers. obs.) consumption rates were low and the highly variable among sites sampled agrees with the previously reported spatial variability in *S*. *salpa* herbivory [5,6]. This variability made it difficult to detect any significant differences among treatments in consumption rates and food choice experiments. Despite the fact that fish abundances are expected to be higher in MPAs, such as Tabarca, it did not lead to enhanced grazing pressure on seagrass meadows [12]. Consumption rates did not reflect any differences between the two locations. These results suggest that nutritional content on its own may not be the only factor driving consumption rates in *P*. *oceanica* meadows. *Sarpa salpa* mobility and broad diet (not only based on seagrasses but other macrophytes, see [7,8,27] could likely explain the variable foraging pattern observed. This may be reflecting an ensemble of complex feeding decisions influenced by chemical and structural macrophytes features, home-range mobility, as well as by temporal and spatial differences in the availability of food resources [11,13,28,42,43], that aimed at optimizing the intake of energy and essential dietary elements within the habitats.

C:N ratios in leaves showed differences between sites and treatments. Overall, in the two localities results suggest that despite the high variability between sites, fertilization did increase nutritional content in leaves, since C:N values in some of the sites were lower in fertilized treatment. In the CIMAR, the average C:N ratios values found in control leaves were 1.20 times higher than in fertilized leaves while in Tabarca there were 1.35 times higher than in fertilized leaves. This prove that fertilization treatment was successful in terms of N content and therefore C:N ratios. By contrast, for epiphytes, fertilization did not appear to increase its nutritional content. Values at CIMAR were similar between sites and treatments and at Tabarca values were only significantly higher in sites five and six but without any apparent difference between treatments. Nevertheless, lower C:N ratios detected in epiphytes compared to leaves confirm their higher nutritional content, as it has been previously reported [7,19,20]. We think that the lack of differences between control and fertilized plots could be probably explained by the fact that fertilization rods were added to the sediment, not directly to the leaves, so nutrients could have been diluted substantially when effluxing the sediment, and before getting to the epiphytes. There is also plausible that other grazers may have influenced on the epiphytic load during the experiment, by removing some epiphytes and therefore making difficult to detect fertilization effects on them.

Growth rates were not different between locations or treatments, but showed high variability between sites. At CIMAR, values recorded were similar between sites and treatments, except for site three which had the lowest recorded value in the fertilized treatment. At Tabarca, site four had higher values than the other sites, being significantly higher in control vs. fertilized treatment. Although fertilization did increase nutritional content in the leaves at some of sites, growth rates seemed to respond to natural variability within the meadow rather than to the fertilization treatment as it was expected [11]. We think that the lack of differences in growth rates between fertilized and control plots may be explained by two possibilities. First, that the experiment may not have been long enough to detect differences on growth rates (some of the past fertilization/growth experiments with *Posidonia* have run for at least a year, see [11]). Second, that the fertilizer level applied was high enough to cause detrimental effects on *Posidonia* growth. In fact, several studies have related seagrass decline to the effects of nutrient excess on plant physiology [44], and with nutrient-induced interactions between plants and other components of the seagrass community, such as macroalgae, epiphytes and herbivores [44,45, 46,47,48,49]. As we reported before in material and methods section, we deliberately applied a high fertilization dose to mimic large, realistic inputs such as from sewage pipes, and we know that the dose we used exceed 18 times the dose recommended, so this theory seems quite plausible. This was also evident during measurements, as we noticed that fertilized shoots were more damaged/degraded than control shoots.

Despite schools of *S*. *salpa* being frequently observed around the plots (Marco-Méndez pers.obs.) and the duration of the experiment (30 days), consumption was low or not detected at some of the sites. Indeed, patterns of herbivory did not follow the expected tendency for higher consumption on fertilized plots but showed a high variability which made it difficult to detect differences between locations, sites, or treatments. However, after performing statistical power analyses, results were at least suggestive of a correct decision when accepting our null hypothesis that herbivory by *S*. *salpa* was not higher in the fertilized plots compared to the non-fertilized plots. The high variability found in our study, concurs with the high spatial variability previously reported for *S*. *salpa* herbivory on *P*. *oceanica* [5,6] which has been attributed to changes in fish abundances and distribution as a result of the interaction among recruitment rates [50], predation effects [51] or overfishing [52]. However, despite fish abundances are expected to be higher in fishing protected areas such as Tabarca, and consequently expected to lead on enhanced grazing pressure over the seagrass meadow [12], consumption rates did not reflected any difference between the two locations. Among other possible factors, studies also suggest that fish schools’ movements across a mosaic of underwater habitats can account for different concentrations in seagrass patches. Indeed, *S*. *salpa*’s home range is of the order of 4 ha [42,43], and mobility across different habitats has been previously documented [3]. In mobile species with comparable home range areas, density area relationships have been associated with either random searching patterns [53] or the use of visual or olfactory cues to find the resources [54]. *Sarpa salpa* juveniles and adults are mid-water visual foragers moving widely within their summer home range, which includes seagrass meadows, rocky substrates and sandy areas. Although large schools of adult *S*. *salpa* are common browsers in *P*. *oceanica*, as we observed in our study, they also feed on a wide range of ‘macroalgae’ and seagrasses [7,8,55,56] and may aim at maintaining a diverse diet to achieve the required nutrients [57] which would lead to highly variable feeding patterns.

*S*. *salpa* feeding choices have been suggested to be mainly driven by nutritional contents in macrophytes [7,28]. However, our findings suggest that nutritional content by itself may not be the only factor driving final consumption rates and that this herbivore may have a wider diet in which other macrophytes features could be also involved [28,29]. Furthermore, herbivores choices can be critically mediated by different levels of secondary metabolites, toughness [30], energy contents [31] or structural carbohydrates (cellulose) affecting food digestibility, and absorption (e.g. [32]) which can vary greatly between different macrophytes species. Indeed, the apparent tolerance of this fish for caulerpenyne [27,58], a secondary metabolite synthesized by species of the genus *Caulerpa* to deter grazing, allow them to feed largely on these species [7,8,27]. Besides structural defenses, the limited literature suggests that dominant species with relatively slow growth rates, such as *P*. *oceanica* [59], are chemically defended whereas other ephemeral pioneer species with faster growth rates [60], show no deterrence [61]. According to this idea, *C*. *nodosa* and other fast-growing species could be less chemically defended and thus more highly grazed on by herbivores than slower growing species like *P*. *oceanica* [7,8].

In this study, the low consumption rates of *P*. *oceanica* leaves detected, even after fertilization, indicates that *S*. *salpa* pressure over *P*. *oceanica* may not be as prominent as it has been reported in other studies [5,6]. This and other studies suggest *S*. *salpa* is often generalist consumers not only dictated by diet nutrient content but by complex interactions between other traits of nutritional quality, habitat heterogeneity within their ample foraging area, and responses to predation risk.

## Supporting Information

S1 Data Growth data. (XLSX)

S2 Data C:N ratio in leaves. (XLSX)

S3 Data C:N ratio in piphytes. (XLSX)

S4 Data Consumption rates. (XLSX)

S5 Data Food choice data. (XLSX)
